# Effect of carbon nanoparticle suspension injection versus indocyanine green tracer in guiding lymph node dissection during radical gastrectomy (FUTURE-01): a randomized clinical trial

**DOI:** 10.1097/JS9.0000000000001873

**Published:** 2024-07-02

**Authors:** Yuan Tian, Yue Pang, Peigang Yang, Shuo Guo, Wenqian Ma, Honghai Guo, Yang Liu, Ze Zhang, Pingan Ding, Tao Zheng, Yong Li, Liqiao Fan, Zhidong Zhang, Dong Wang, Xuefeng Zhao, Bibo Tan, Yu Liu, Qun Zhao

**Affiliations:** a Third Surgery Department, The Fourth Hospital of Hebei Medical University; b Hebei Key Laboratory of Precision Diagnosis and Comprehensive Treatment of Gastric Cancer; c Big Data Analysis and Mining Application for Precise Diagnosis and Treatment of Gastric Cancer, Hebei Provincial Engineering Research Center; d Department of Endoscopy, The Fourth Hospital of Hebei Medical University, Shijiazhuang, People’s Republic of China

**Keywords:** carbon nanoparticle suspension injection, indocyanine green, lymph node dissection, lymphatic navigation technique, radical gastrectomy

## Abstract

**Background:**

Carbon nanoparticle suspension injection (CNSI) and indocyanine green (ICG) have both been applied intraoperatively to facilitate lymphatic mapping and postoperatively to sort lymph nodes (LNs) in gastric cancer patients. However, no study has compared the two tracers in gastric cancer patients.

**Materials and methods:**

This prospective randomized controlled trial was conducted from January 2022 to March 2023. Patients with potentially resectable gastric cancer (cT1-4a N0/+ M0) were randomized to the CNSI or ICG group.

**Results:**

This study enrolled 96 patients. Ninety patients were in the modified intention-to-treat population, including 46 patients [32 males and 14 females; mean (SD) age, 57.4 (9.4) years] in the CNSI group and 44 patients [31 males and 13 females; mean (SD) age, 60.8 (8.8) years] in the ICG group. The mean (SD) number of retrieved LNs was 69.8 (21.9) and 53.6 (17.2) in the CNSI and ICG groups, respectively (*P*<0.001). The mean (SD) number of retrieved micro-LNs was 19.9 (13.3) and 11.6 (9.9) in the CNSI and ICG groups, respectively (*P*=0.001). The mean (SD) number of metastatic LNs was 8.1 (11.9) and 5.2 (9.2) in the CNSI and ICG groups, respectively (*P*=0.19).

**Conclusions:**

Compared with ICG, CNSI can increase the number of LNs detected, especially micro-LNs. Both tracers have high diagnostic value for detecting metastatic LNs. CNSI-guided lymphography may be a superior method for improving the accuracy of LN dissection.

## Introduction

Highlights

- Preoperative endoscopic injection of carbon nanoparticle suspension injection (CNSI) can improve the number of lymph nodes (LNs) detected.
- Both CNSI and indocyanine green (ICG) have high diagnostic value in metastatic LNs.
- The cost of CNSI in clinical application is lower than that of ICG, and it has better clinical promotion value.

High-quality lymphadenectomy during radical gastrectomy can improve the prognosis of patients with gastric cancer^1–3^, and the detection of more LNs after surgery facilitates accurate pathological N staging^4,5^. However, avoiding insufficient or blindly extended lymphadenectomy has become a challenge for surgeons.

The development of laparoscopic and robotic surgery has led to advancements in minimally invasive surgery for gastric cancer^6^. LN tracing technology has been widely used in clinical practice^7^. The combination of minimally invasive surgery and LN tracing techniques is an important method for improving the quality of lymphadenectomy and correcting the bias of postoperative N staging.

In recent years, different dyes and tracers have been studied to observe LN drainage from primary tumors. For gastric cancer patients, CNSI and ICG have been used as two novel LN tracers to facilitate intraoperative lymphatic mapping and postoperative classification of LNs in extracted pathological specimens^8–13^. Carbon nanoparticle suspensions are composed of activated carbon particles whose diameter is between the vascular endothelial cell gap and the lymphatic endothelial cell gap. These particles do not enter the blood but directly invade the lymphatic vessels and accumulate in LNs, and the stained LNs are visible to the naked eye. After binding to serum albumin, ICG emits infrared fluorescence when it is stimulated by infrared light. Fluorescent LNs can be observed with a near-infrared (NIR) fluorescence imaging system.

The choice between the two tracers is mostly based on the surgeon’s personal preference or the equipment at the institution. There is still a lack of prospective randomized controlled clinical studies comparing the two tracers in depth. Therefore, this study aimed to compare and evaluate the efficacy, feasibility, and safety of CNSI and ICG in laparoscopic or robot-assisted radical gastrectomy. Furthermore, this investigation provides a reference for standardized LN tracing technology in radical gastrectomy.

## Materials and methods

The FUTURE-01 trial was a phase 3 prospective randomized open-label clinical trial conducted between January 20, 2022, and March 9, 2023. The primary endpoint was the number of LNs retrieved. The secondary endpoints were the diagnostic value of stained (fluorescent) LNs, surgical results, postoperative recovery course, 3-year disease-free survival, 3-year overall survival, and recurrence pattern within 3 years. The trial protocol was approved by the ethics committee (trial protocol in Supplement 1, Supplemental Digital Content 1, http://links.lww.com/JS9/C964)^14^. All participants provided written informed consent before enrollment. This trial followed the Consolidated Standards of Reporting Trials (CONSORT, Supplemental Digital Content 2, http://links.lww.com/JS9/C965) reporting guidelines^15^.

### Eligibility criteria

The inclusion criteria were as follows: aged 18–75 years, histologically proven gastric adenocarcinoma on biopsy, proven clinical stage of cT1-4a N0/+ M0 by ultrasound endoscopy, enhanced computed tomography/MRI or diagnostic laparoscopy according to the TNM classification of the American Joint Committee on Cancer (eighth edition), preoperative Eastern Cooperative Oncology Group score of 0 or 1, and preoperative American Society of Anesthesiologists score of Ⅰ–III. The exclusion criteria were as follows: pregnancy or lactation, history of gastrectomy, endoscopic mucosal resection, or endoscopic submucosal dissection, enlarged or bulky (larger than 3 cm in diameter) regional LNs according to preoperative imaging, tumor invasion of the duodenum or esophagus, and Borrmann type IV gastric cancer. The detailed eligibility criteria are described in the SDC trial protocol (Supplemental Digital Content 1, http://links.lww.com/JS9/C964).

### Randomization and blinding

Patients who met the inclusion criteria and were eligible for radical oncosurgery were randomly assigned to the CNSI group or the ICG group at a 1:1 ratio by an investigator (Y.P.). A random number table generated by the data manager was used for random allocation. However, blinding the surgeons or participants was impossible.

### Interventions

#### Preoperative measures

In the CNSI group, the patients were given injections of CNS (50 mg/dose) produced by Chongqing Lummy Pharmaceutical Co. Ltd, in the endoscopy division 1 day before surgery. The CNS was injected submucosally at four points 0.5–1 cm from the tumor edge under endoscopy. The optimized dose for each point was approximately 0.25 ml (Video 1, Supplemental Digital Content 3, http://links.lww.com/JS9/C966).

In the ICG group, ICG (25 mg/dose) produced by Dandong Yichuang Pharmaceutical was marked in the endoscopy division 1 day before surgery and injected submucosally at four points 0.5–1 cm from the tumor edge under endoscopy. The optimized dose for each point was approximately 0.5 ml (Video 2, Supplemental Digital Content 4, http://links.lww.com/JS9/C967). A designated, experienced endoscopic specialist performed both procedures.

#### Surgery

The same group of surgeons performed lymphadenectomies. Laparoscopic exploration is required to rule out peritoneal implantation metastases and cytological positivity for abdominal exfoliation. In the CNSI group, radical gastrectomy was performed with a conventional laparoscopic surgical system or a Da Vinci XI robotic surgical system (Intuitive Surgical). In the ICG group, the operation was performed with the NOVADAQ fluorescent laparoscopic surgical system or Da Vinci XI robotic surgical system. Distal or total gastrectomy was selected according to the tumor location and performed according to the Japanese Gastric Cancer Treatment Guidelines^16^.

CNSI and ICG were used for quality control in LN dissection, and residual LNs were dissected (Videos 3, Supplemental Digital Content 5, http://links.lww.com/JS9/C968 and 4, Supplemental Digital Content 6, http://links.lww.com/JS9/C969). Although stained LNs were occasionally detected outside the planned dissection area (e.g., stations 8p and 14v), excessive dissection beyond the scope of D2 lymphadenectomy was not performed.

### Outcome measurements

The surgeon (Y.P. or H.-H.G.) appointed to perform the LN dissection had thorough knowledge of LNs and abundant experience. The time taken for LN sorting was accurately recorded. In the CNSI group, the LNs were sorted under direct vision (Video 5, Supplemental Digital Content 7, http://links.lww.com/JS9/C970), while in the ICG group, the LNs were sorted with the aid of a direct NIR imaging system (Video 6, Supplemental Digital Content 8, http://links.lww.com/JS9/C971). LNs were classified and sent to the pathology department for detection according to the station, staining condition, and maximum diameter (>2 or ≤2 mm). A micro-LN was defined as an LN with a maximum diameter of less than 2 mm^17^.

Morbidity and mortality were assessed at 30 days after surgery. Postoperative complications were graded according to the Clavien—Dindo classification^18^.

### Sample size and statistical analysis

The sample size of this study was calculated using PASS (version 11; NCSS LLC Ltd). All analyses were two-sided, with α=0.05 and β=0.80. According to our previous study^10,19^, the expected mean number of LNs in the CNSI group was 56.93. According to two additional previous studies^11,13^, the expected mean number of LNs in the ICG group was 50.52. The total sample size was 96 (48 per group) after accounting for a 10% drop-out rate in each group. The planned recruitment period was 1 year.

Patients who were randomized and those who met the postrandomization exclusion criteria were excluded from the modified intention-to-treat population. Descriptive data are presented herein as the mean±SD, while categorical data are presented as the number and percentage. The independent-sample *t* test or nonparametric test was used to analyze continuous data, and the *χ* ^2^ test or Fisher’s exact test was used to analyze categorical variables. SPSS 26.0 statistical software (IBM) was used for the statistical analysis. *P* value <0.05 was considered to indicate a statistically significant difference.

## Results

### Baseline characteristics

**Figure 1:**
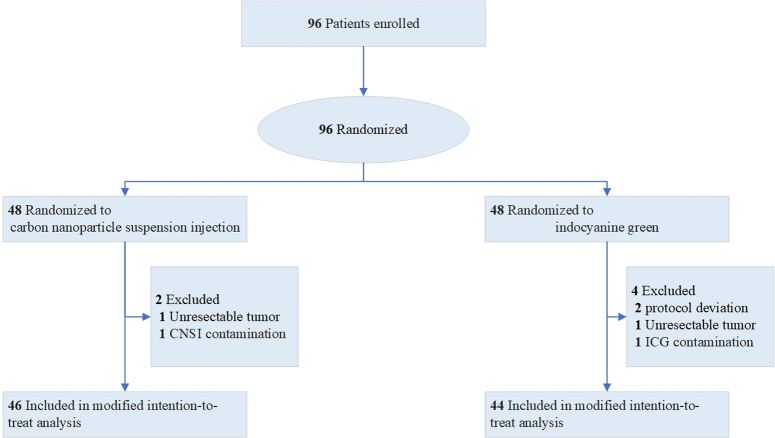
Consolidated Standards of Reporting Trials (CONSORT) flowchart.

A total of 96 patients were randomized to either the CNSI group or the ICG group between January 20, 2022, and March 9, 2023. Of those, 48 patients were in the CNSI group, and 48 patients were in the ICG group. Six patients were excluded (two due to protocol deviation, two due to unresectable tumors, one due to CNSI contamination, and one due to ICG contamination). CNSI-guided lymphadenectomy was performed for 46 patients, and ICG tracer-guided lymphadenectomy was performed for 44 patients. These populations were defined as the modified intention-to-treat population. A CONSORT flow diagram of this randomized controlled trial (RCT) is shown in Figure 1.

Table 1 shows the patient demographic characteristics. No significant differences were observed between the two groups. There were no differences between the two groups in terms of tumor location, tumor size, tumor type, or tumor stage distribution. The tumor characteristics were well-balanced between the groups.

**Table 1:** Patient demographic and tumor characteristics in the carbon nanoparticle suspension injection and indocyanine green groups.

	*N* (%)		
Characteristics	CNSI (*n*=46)	ICG (*n*=44)	*P*
Age, years	57.4 (9.4)	60.8 (8.8)	0.08
BMI	24.9 (3.9)	25.2 (3.3)	0.81
Sex			
Male	32 (69.9)	31 (70.5)	0.93
Female	14 (30.4)	13 (29.5)	
ECOG performance status			
0	37 (80.4)	33 (75)	0.54
1	9 (19.6)	11 (25)	
Tumor location			
Upper	8 (17.4)	12 (27.3)	0.21
Middle	18 (39.1)	10 (22.7)	
Lower	20 (34.5)	22 (50)	
Histologic type (Lauren classification)			
Intestinal	7 (15.2)	8 (18.2)	0.92
Diffuse	20 (43.5)	19 (43.2)	
Mixed	19 (41.3)	17 (38.6)	
Surgical procedure			
Distal gastrectomy	29 (63)	28 (63.6)	0.95
Total gastrectomy	17 (37)	16 (36.4)	
Reconstruction method			
Billroth II	29 (63)	28 (63.6)	0.95
Roux-en-Y	17 (37)	16 (36.4)	
Surgical approach			
Laparoscopic	13 (28.3)	10 (22.7)	0.55
Robotic	33 (71.7)	34 (77.3)	
Size, cm			
≤3	22 (47.8)	29 (65.9)	0.08
>3	24 (52.2)	15 (34.1)	
cT stage			
cT1	15 (32.6)	11 (25)	0.73
cT2–cT3	10 (21.7)	11 (25)	
cT4	21 (45.7)	22 (50)	
cN stage			
cN0	17 (37)	18 (40.9)	0.72
cN1	15 (32.6)	12 (27.3)	
cN2	14 (30.4)	13 (29.5)	
cN3	0	1 (2.3)	
pT stage			
pT1	17 (37)	14 (31.8)	0.85
pT2–pT3	6 (13)	7 (15.9)	
pT4	23 (50)	23 (52.3)	
pN stage			
pN0	22 (47.8)	22 (50)	0.51
pN1	2 (4.3)	4 (9.1)	
pN2	6 (13)	9 (20.5)	
pN3a	6 (13)	4 (9.1)	
pN3b	10 (21.7)	5 (11.4)	
p stage			
I	18 (39.1)	17 (38.6)	0.99
II	8 (17.4)	8 (18.2)	
III	20 (43.5)	19 (43.2)	
Nerve invasion			
Negative	21 (45.7)	20 (45.5)	0.98
Positive	25 (54.3)	24 (54.5)	
Lymphovascular invasion			
Negative	36 (78.3)	38 (86.4)	0.32
Positive	10 (21.7)	6 (13.6)	

BMI, body mass index (calculated as weight in kilograms divided by height in meters squared); ECOG, Eastern Cooperative Oncology; CNSI, carbon nanoparticle suspension injection; ICG, indocyanine green.

### Lymph nodes dissection

The mean (SD) number of retrieved LNs in the CNSI group was 69.8 (21.9), which was significantly greater than the number of retrieved LNs in the ICG group [mean (SD), 53.6 (17.2); *P*<0.001]. However, there was no significant difference in the number of patients with a history of total gastrectomy, intestinal adenocarcinoma, or stage cT2-3, pT1-3, or c/pN0 disease between the two groups (Table 2).

**Table 2:** Number of retrieved lymph nodes in the carbon nanoparticle suspension injection and indocyanine green groups

	Mean (SD)		
Variables	CNSI (*n*=46)	ICG (*n*=44)	*P*
Total number of LNs retrieved BMI	69.8 (21.9)	53.6 (17.2)	<0.001
≤24	62.5 (20.5)	50.9 (13.7)	0.05
>24	75.9 (21.7)	55.2 (18.9)	<0.001
Sex			
Male	68.5 (20)	54.2 (17.4)	0.004
Female	72.6 (26.5)	52.2 (17.3)	0.02
Surgical procedure			
Distal gastrectomy	68.6 (24)	49.1 (15.1)	<0.001
Total gastrectomy	71.8 (18.5)	61.6 (18.2)	0.12
Histologic type (Lauren classification)			
Intestinal	65.3 (11.6)	54.7 (16.5)	0.17
Diffuse	76.8 (26.5)	54.3 (15.6)	0.003
Mixed	64.1 (18.1)	52.3 (20.1)	0.08
Surgical approach			
Laparoscopic	62.6 (20.2)	46.1 (6.1)	0.02
Robotic	72.6 (22.3)	55.8 (18.7)	<0.001
cT stage			
cT1	72.7 (24.9)	49.5 (18.5)	0.01
cT2–cT3	57 (19.4)	55.8 (18.3)	0.87
cT4	75.4 (18.6)	55 (16.1)	<0.001
cN stage			
cN0	63.5 (15.7)	54.3 (19.4)	0.13
cN+	73.4 (24.4)	53.1 (15.7)	<0.001
pT stage			
pT1	62 (15.3)	52.2 (18.6)	0.12
pT2–pT3	57.1 (17.9)	46.6 (11.9)	0.26
pT4	78.8 (23.8)	56.7 (17.5)	<0.001
pN stage			
pN0	61.8 (14.3)	53.7 (18.5)	0.11
pN+	77 (25.3)	53.5 (16.1)	<0.001
p stage			
I	65 (14.4)	51.8 (17.9)	0.02
II	49.7 (10.7)	54.8 (17.9)	0.49
III	82.1 (23.7)	54.6 (16.9)	<0.001

BMI, body mass index (calculated as weight in kilograms divided by height in meters squared); CNSI, carbon nanoparticle suspension injection; ICG, indocyanine green; LN, lymph nodes.

**Figure 2:**
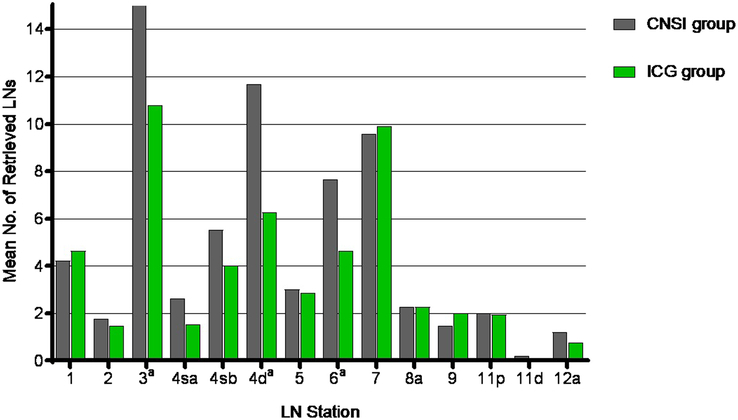
Retrieved lymph nodes (LNs) according to the LN station. CNSI, carbon nanoparticle suspension injection; ICG, indocyanine green. ^a^ *P* value <0.05 between groups.

**Figure 3:**
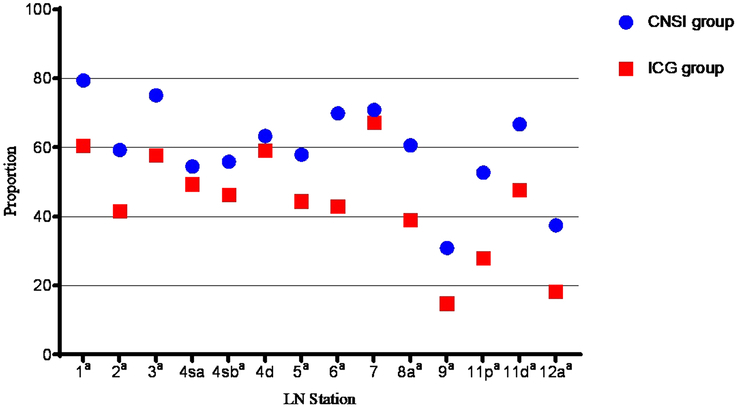
Ratio of black-stained/fluorescent lymph nodes (LNs) according to LN station. CNSI, carbon nanoparticle suspension injection; ICG, indocyanine green. ^a^ *P* value <0.05 between groups.

The mean (SD) number of detected LNs per station in the CNSI group was 4.9 (1.6), which was higher than the mean number of detected LNs per station in the ICG group [3.8 (1.2) LNs per station; *P*<0.001]. In particular, there were statistically significant differences at stations 3, 4d, and 6 (Fig. 2). The proportion of black-stained LNs in the CNSI group was 66.1% (2123/3210), which was greater than the proportion of fluorescent LNs in the ICG group (51.8%, 1222/2360; *P*<0.001). There were significant differences at stations 1, 2, 3, 4 sb, 5, 6, 8a, 9, 11p, 11d, and 12a (*P*<0.05, Fig. 3).

**Figure 4:**
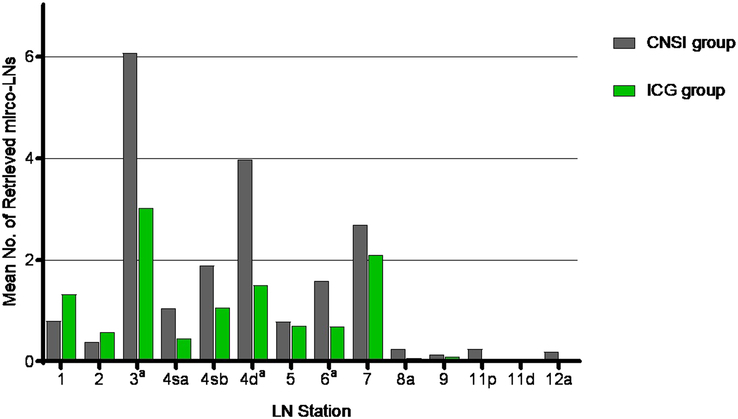
Microlymphs (micro-LNs) retrieved according to LN station. CNSI, carbon nanoparticle suspension injection; ICG, indocyanine green. ^a^ *P* value <0.05 between groups.

**Figure 5:**
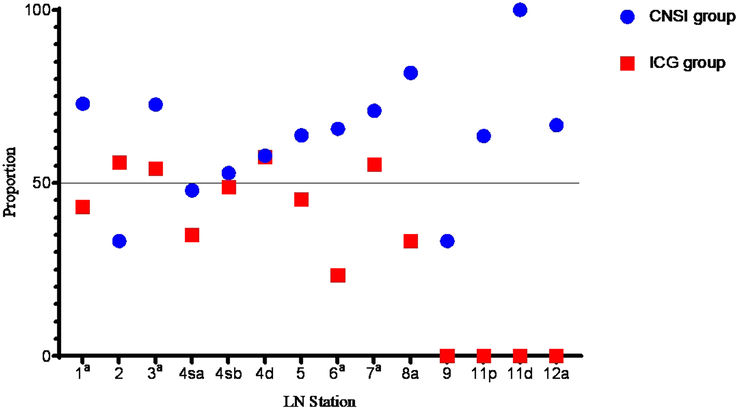
Ratio of black-stained/fluorescent microlymph nodes (micro-LNs) according to the LN station. CNSI, carbon nanoparticle suspension injection; ICG, indocyanine green. ^a^ *P* value <0.05 between groups.

The mean (SD) number of retrieved micro-LNs in the CNSI group was 19.9 (13.3), which was significantly greater than the mean number of retrieved micro-LNs in the ICG group [mean (SD), 11.6 (9.9); *P*=0.001]. A mean (SD) of 2 (1.1) micro-LNs per station was detected in the CNSI group, which was greater than that detected in the ICG group [1.4 (0.7) LNs per station; *P*<0.001]. There were statistically significant differences at stations 3, 4d, and 6 (Fig. 4). The proportion of black-stained micro-LNs in the CNSI group was 63.9% (587/918), which was greater than the proportion of fluorescent micro-LNs in the ICG group (49.2%, 252/512; *P*<0.001). There were significant differences at stations 1, 3, 6, and 7 (*P*<0.05, Fig. 5).

### Lymph nodes metastasis

The mean (SD) number of metastatic LNs in the CNSI group [8.1 (11.9)] was not significantly higher than that in the ICG group [5.2 (9.2); *P*=0.19]. There were no significant differences at any individual LN station (SDC, Supplementary Table 1, Supplemental Digital Content 9, http://links.lww.com/JS9/C972).

Twenty-five of the 46 patients in the CNSI group (54.3%) and 24 of the 44 patients in the ICG group (54.5%) had LN metastasis. The sensitivity of CNSI-guided and ICG-guided lymphography for detecting metastatic LNs was 72.1 and 42.8%, respectively; the specificity was 34.6 and 47.3%, respectively; the positive predictive value was 12.7 and 8%, respectively; and the negative predictive value was 90.4 and 88.5%, respectively (SDC, Supplementary Table 2, Supplemental Digital Content 10, http://links.lww.com/JS9/C973).

Micro-LN metastasis occurred in 20 of 46 patients in the CNSI group and eight of 44 patients in the ICG group. The sensitivity of CNSI-guided and ICG-guided lymphography for detecting metastatic micro-LNs was 70.9 and 40.6%, respectively; the specificity was 36.6 and 50.2%, respectively; the positive predictive value was 7.5 and 5.2%, respectively; and the negative predictive value was 94.6 and 92.7%, respectively (SDC, Supplementary Table 3, Supplemental Digital Content 11, http://links.lww.com/JS9/C974).

### Postoperative evaluation

No significant differences between the CNSI and ICG groups were observed in terms of intraoperative blood loss [mean (SD), 38.3 (41.2) ml vs. 36.8 (37.8) ml; *P*=0.14] or operative time [mean (SD), 201.4 (33.4) min vs. 212.0 (37.4) min; *P*=0.52]. The postoperative recovery process was comparable in both groups. There were no significant differences in the time to first flatus, time to first liquid intake, or postoperative hospital stay between the two groups. No significant differences were found between the CNSI and ICG groups regarding the incidence of postoperative complications within 30 days after surgery [five of 46 patients (10.9%) vs. five of 44 patients (11.4%); *P*=0.94] or the severity of postoperative complications. The mean (SD) duration of LN dissection assisted by the direct NIR imaging system was significantly longer in the ICG group [19.4 (10.7)] than in the CNSI group [37.1 (12.8); *P*<0.001] (Table 3).

**Table 3:** Surgical outcomes and morbidity in the carbon nanoparticle suspension injection and indocyanine green groups.

	CNSI group (*n*=46)	ICG group (*n*=44)	
	Mean±SD/*N* (%)	Mean±SD/*N* (%)	*P*
Surgical outcomes			
Blood loss (ml)	38.3±41.2	36.8±37.8	0.14
Surgical time (min)	146.5±48.2	151.3±50.1	0.52
Time to first exhaust (days)	2.2±0.9	2.1±0.9	0.76
Time to postoperative liquid diet intake (days)	4.0±1.8	3.9±1.8	0.63
Postoperative hospitalization (days)	6.9±1.2	6.8±1.4	0.79
Postoperative LN anatomy duration (min)	19.4±10.7	37.1±12.8	<0.001
Morbidity			
Postoperative complication	5 (10.9)	5 (11.4)	0.94
Anastomotic leakage	0	1（2.3）	0.30
Abdominal infection	1(2.2)	0	0.33
Lymphatic leakage	1(2.6)	0	0.33
Gastroparesis	0	2(4.5)	0.14
Intestinal obstruction	2(4.3)	2(4.5)	0.96
Serious pneumonia	1(2.2)	1(2.3)	0.98
Mortality	0	0	1.00
Clavien—Dindo classification			0.77
I	1(2.2)	2(4.5)	
II	3(6.5)	2(4.5)	
III	1(2.2)	1(2.3)	
IV	0	0	
V	0	0	

CNSI, carbon nanoparticle suspension injection; ICG, indocyanine green; LN, lymph nodes.

One patient in each group was excluded due to contamination of abdominal adipose tissue caused by an excessively deep endoscopic submucosal injection (SDC, Supplementary Figure 1, Supplemental Digital Content 12, http://links.lww.com/JS9/C975). No complications of CNSI or ICG injection occurred in either group. Due to the COVID-19 prevention and control policy, two patients in the ICG group underwent surgery 7 days after endoscopic ICG injection. However, the ICG fluorescence was localized only to the gastric wall, and no fluorescence was detected in the LNs.

## Discussion

The most important steps in radical gastrectomy for gastric cancer are adequate LN dissection and surgical margin determination. The widespread use of laparoscopic and robotic surgery has weakened the sense of touch during surgery, making it difficult to judge the tumor location and resection margin. The application of LN tracers guides navigating the lymphatic system and effectively solves the problem of surgical margins. However, previous studies have analyzed single LN tracers or multiple combined applications, and comparative analyses are lacking^20–24^. Our study is the first to report the results of a prospective clinical RCT of CNS and ICG.

The methods used for injecting LN tracers mainly include preoperative submucosal injection and intraoperative subserosal injection. The injection of tracers into tissue surrounding subserosal tumors is affected by the intraoperative judgment of the tumor location, which may lead to a reduction in the accuracy of injection and lymphatic tracing. However, injecting the lesser curvature and the greater curvature of the stomach lacks the specificity of the lymphatic drainage pattern from the tumor, which reflects the pattern of lymphatic drainage from the stomach. Therefore, we used a uniform submucosal injection as the standard.

Our results indicated that the number of LNs retrieved in the CNSI and ICG groups was 69 and 53, respectively, which is greater than the basic requirement for the number of LNs retrieved^25,26^. The number of LNs retrieved in the CNSI group was greater than that in a previous study^19^, which may indicate that submucosal CNSI can increase the number of LNs retrieved compared with subserosal CNSI, thus providing a basis for further prospective research. The number of LNs retrieved in the ICG group was similar to that in previous studies^11–13,27^ but lower than that in the CNSI group, which may be related to the green fluorescence imaging mode of the Da Vinci robot, which is mostly used to determine tissue blood supplies. In addition, the sorting of LNs in the CNSI group could be performed directly under the naked eye, while in the ICG group, assistance from a fluorescence lens was needed, which increased not only the material consumption of the procedure but also the degree of visual fatigue of the surgeon, which is one of the reasons for the retrieval of fewer LNs. Our results also indicated that the duration of LN dissection assisted by the direct NIR imaging system was significantly longer in the ICG group than in the CNSI group.

CNSI-guided lymphadenectomy is more advantageous for patients with a history of distal gastrectomy or poorly differentiated and advanced gastric cancer with LN metastasis. Additionally, more LNs were detected in the CNSI group at stations 3, 4d, and 6, which may indicate that the advantage of CNSI is more obvious when the tumor is located in the lesser curvature or the lower part of the stomach.

Although the number of LNs retrieved in the CNSI group was greater than that in the ICG group, there was no significant difference in the number of metastatic LNs between the two groups. This means that more negative LNs were detected in the CNSI group. Previous studies have shown that increasing the number of negative LNs examined can improve the prognosis, which may be related to the presence of occult metastasis among negative LNs^28–32^. For gastric cancer patients without LN involvement, an LN retrieval number of ≥22 is helpful for prolonging overall survival, especially for individuals with stage T4 disease. The total number of LNs retrieved is an independent prognostic factor for this population of patients^33^.

In some studies, metastatic LNs have been defined as those with a diameter >10 mm along the short axis or a short/long axis diameter ratio of 0.75 on computed tomography^34,35^. The diagnosis of bulky LNs is emphasized in the clinic, and it is easy to ignore micro-LNs. It is often believed that the retrieval of micro-LNs increases the clinical workload and that micro-LNs have almost no possibility of metastasis. However, the results of this study showed the presence of metastases in micro-LNs. Therefore, examining micro-LNs cannot only increase the total number of LNs examined but also increase the number of positive LNs examined, thereby improving the accuracy of postoperative pathological N staging and correcting the bias of N staging^36,37^. The application of CNSI can effectively help surgeons harvest more micro-LNs.

We found no differences in the amount of bleeding, operative time, or postoperative recovery time between the two lymphatic navigation techniques used for radical gastrectomy. The incidence of postoperative complications did not increase in either group compared with that in conventional radical gastrectomy^38,39^, and no complications related to the injection of lymphatic tracers occurred in either group. This indicates that the safety of the two lymphatic navigation techniques in radical gastrectomy is reliable.

### Limitations

This study has several limitations. First, compared with ICG, CNSI was found to have an advantage in terms of the total number of LNs retrieved; however, it remains unclear whether this advantage can translate into improved long-term survival, and long-term follow-up data are still needed. There have been no reports on improvements in the survival of patients with gastric cancer after radical gastrectomy with an LN tracer. Second, this study was conducted at a high-volume medical center with extensive experience in the surgical treatment of gastric cancer, and confirmation from a multicenter study is still lacking. Third, this RCT did not include patients who received neoadjuvant therapy. Although the role of CNSI and ICG in radical gastrectomy after neoadjuvant therapy has been studied previously^9,40^, the comparative effect of the two tracers in surgery after neoadjuvant therapy needs to be further explored.

## Conclusions

This is the first RCT to demonstrate that CNSI can improve the number of retrieved LNs, the number of retrieved micro-LNs, and the accuracy of pathological N staging compared with ICG. Both CNSI and ICG have high diagnostic value for detecting metastatic LNs. CNSI is less expensive to use, does not require fluorescence systems, and is easier to apply in less-developed health systems or in hospitals that do not have access to the most technologically advanced methods. Thus, CNSI-guided lymphography may be a better method for improving the accuracy of LN dissection.

## Ethical approval

The present study was approved by the Ethical Review Committee of Hebei Medical University (Shijiazhuang, China) (No. 2021185).

## Consent

Written informed consent was obtained from the patient for publication and any accompanying images.

## Source of funding

This study was supported by the Cultivating Outstanding Talents Project of Hebei Provincial Government Fund, No. 2019012; Hebei Public Health Committee County-Level Public Hospitals Suitable Health Technology Promotion and Storage Project, No. 2019024; Hebei Medical University Education and Teaching Research Project, No. 2020CGPY-12, No. 2020CHYB-23; Medical Science Research Project of Hebei Province (20230121).

## Author contribution

Q.Z. and Y.T. designed the study; Y.T. collected the patient data, and drafted the paper; P.Y., H.G., Y.L., Z.Z., P.D., and T.Z. participated in the design of the study and edited the final paper. Y.T., S.G., W.M., Y.L. (Yang Liu), L.F., Z.Z., D.W., X.Z., B.T., and Y.L. (Yu Liu) analyzed and interpreted the data; Q.Z. contributed to the critical revision of the manuscript for important intellectual content and supervised this study. All authors read and approved the paper for publication.

## Conflicts of interest disclosure

The authors declare no conflicts of interest.

## Research registration unique identifying number (UIN)

1. Name of the registry: A Prospective Randomized Controlled Study of the Efficacy of Carbon Nanoparticles Versus Indocyanine Green in Robotic or Laparoscopic Gastrectomy.
2. Unique identifying number or registration ID: NCT05229874.
3. Hyperlink to your specific registration (must be publicly accessible and will be checked):https://clinicaltrials.gov/study/NCT05229874?cond=NCT05229874&rank=1.

## Guarantor

Qun Zhao.

## Data availability statement

The datasets used and/or analyzed during the current study are available from the corresponding authors upon reasonable request.

## Provenance and peer review

This study was not commissioned and was externally peer-reviewed.

## Supplementary Material
