# Supplementary material for: Effect of carbon nanoparticle suspension injection versus indocyanine green tracer in guiding lymph node dissection during radical gastrectomy (FUTURE-01): a randomized clinical trial
Source: Int J Surg. 2024 Jul 2;111(1):609–16. doi: 10.1097/JS9.0000000000001873 (PMC11745718; doi:10.1097/JS9.0000000000001873)
Supplement: Supplementary file 1 [file js9-111-0609-s001.docx]

**A randomized controlled clinical trial of carbon nanoparticles versus indocyanine green guided lymph node mapping in radical gastrectomy for gastric cancer**

Version: 1.0

Protocol Date: 2021.10.01

Protocol Leader: Qun Zhao

Sites: The Fourth Hospital of Hebei Medical University

Directory

[Protocol brief 1](#_Toc75527954)

[1、Background 4](#_Toc75527955)

[2、Purpose, content and significance of the research 5](#_Toc75527956)

[3、Implementation protocol 5](#_Toc75527957)

[**Study design** 5](#_Toc75527958)

[**Technical Route** 7](#_Toc75527959)

[**Implement** 7](#_Toc75527960)

[（1）Inclusion criteria 7](#_Toc75527961)

[（2）Exclusion criteria 8](#_Toc75527962)

[（3）Screening, enrollment phase 8](#_Toc75527963)

[（4）Treatment options after enrollment 8](#_Toc75527964)

[（5）Laparoscopic exploration 9](#_Toc75527965)

[（6）Lymph node dissection norms 9](#_Toc75527966)

[（7）Lymph node taking specifications 9](#_Toc75527967)

[（8）Pathology report 11](#_Toc75527967)

[（9）Surgical records 11](#_Toc75527967)

[（10）Surgical complications 11](#_Toc75527967)

[（11）Surgery, lymph node material control 11](#_Toc75527968)

[（12）Ethical requirements and informed consent of the subject 12](#_Toc75527968)

[（13）Progress and completion time of clinical trials 12](#_Toc75527969)

[（14）Retention of data 12](#_Toc75527970)

[References 12](#_Toc75527971)

# PROTOCOL BRIEF

| Research topic | | A randomized controlled clinical trial of carbon nanoparticles versus indocyanine green guided lymph node mapping in radical gastrectomy for gastric cancer |  |
| --- | --- | --- | --- |
| Veesion | | 1.0 |  |
| Site | | The Fourth Hospital of Hebei Medical University |  |
| Research object | | Patients undergoing radical gastric cancer |  |
| study  objective | Main purpose | Number of lymph nodes detected after surgery |  |
|  | Secondary purposes | Lymphatic staining (fluorescence) rate, lymph node staining rate of each group, staining (fluorescence) lymph node positive rate, staining (fluorescence) lymph node negative rate, non-stained (fluorescent) lymph node positive rate, non-stained (fluorescent) lymph node negative rate, lymph node metastasis rate, number of lymph node metastasis, complication rate and mortality within 30 days, 3-year DFS, 3-year OS, 3-year recurrence pattern, intraoperative blood loss, postoperative lymph node sorting time, postoperative recovery (exhaust time, intake and liquid feeding time, discharge time) |  |
| Number of patients planned to be enrolled | | 96 |  |
| researcher | | Qun Zhao |  |
| Site | | The Fourth Hospital of Hebei Medical University |  |
| Patient screening criteria | | Inclusion Criteria:  1. Age 18 to 75 years  2. Primary gastric adenocarcinoma confirmed by endoscopic biopsy pathology (papillary, tubular, mucinous, ring cell or hypodifferentiation)  3. According to the American Joint Committee on Cancer (AJCC) Cancer Staging Manual Eighth Edition, clinical stages cT1-4a, N 0/+, M0 at preoperative evaluation  4. No distant metastasis, no direct invasion of pancreas, spleen and other adjacent organs in preoperative examination.  5. Eastern Collaborative Oncology Group Scale (ECOG) 0 or 1 score  6. American Society of Anesthesia Score (ASA) GRADE I, II, or III  7. Sign a written informed consent form  Exclusion Criteria:  1. Women during pregnancy or lactation  2. Severe mental disorders  3. History of previous upper abdominal surgery (except laparoscopic cholecystectomy)  4. History of previous gastrectomy, endoscopic mucosal resection or endoscopic submucosal dissection  5. Preoperative imaging shows enlarged or enlarged regional lymph nodes with a diameter of more than 3 cm  6. Lesions invade the esophagus or duodenum  7. Borrmann type IV gastric cancer  8. History of other malignant tumors in the past five years  9. Previous history of neoadjuvant chemotherapy or radiotherapy  10. History of unstable angina or myocardial infarction within the past six months  11. History of cerebrovascular accident within the past six months  12. History of continuous and systematic use of corticosteroids within 1 month  13. Simultaneous surgical requirements for other diseases  14. Emergency surgery due to gastric cancer complications (bleeding, obstruction or perforation).  15.FEV1 < 50% of the predicted value. |  |
| End study of standard of care | | 1. The patient has completed the treatment prescribed by the protocol; 2. Unwanted treatment in the event of an adverse event (AE) or serious adverse event (SAE); 3. occurrence of unpredictable and unacceptable adverse drug reactions; 4. other circumstances in which the investigator deems it necessary to withdraw from the study; | |
| Shedding/rejection criteria | | 1. Failure to undergo radical gastric resection; 2. failure to administer drug injections in accordance with the dosage, method and drug injection prescribed in this study protocol; |  |
| Exit study criteria | | 1. Allergic reactions  2. The patient requests to withdraw |  |
| Dosing regimen | | In the CNSI group, the patients are given injections of CNSI (50 mg/dose) produced by Chongqing Lummy Pharmaceutical Co., Ltd. in the endoscopy division 1 day before surgery. CNSI is injected submucosally at 4 points (proximal side, distal side, and left and right sides) 0.5-1 cm from the tumor edge under endoscopy. The optimized dose for each point is approximately 0.25 ml.  For those who were randomly assigned to the ICG group, ICG (25 mg/dose) produced by Dandong Yichuang Pharmaceutical is marked in the endoscopy division 1 day before surgery and is injected submucosally at 4 points (proximal side, distal side, and left and right sides) 0.5-1 cm from the tumor edge under endoscopy. The optimized dose for each point is approximately 0.5 ml.Both procedures are performed by a designated, experienced endoscopic specialist. |  |
| .The main efficacy indicators | | Number of lymph nodes detected after surgery |  |
| Secondary efficacy measures | | Lymphatic staining (fluorescence) rate, lymph node staining rate of each group, staining (fluorescence) lymph node positive rate, staining (fluorescence) lymph node negative rate, non-stained (fluorescent) lymph node positive rate, non-stained (fluorescent) lymph node negative rate, lymph node metastasis rate, complication rate and mortality within 30 days, 3-year DFS, 3-year OS, 3-year recurrence pattern, intraoperative blood loss, postoperative lymph node sorting time, postoperative recovery (exhaust time, intake time, discharge time) |  |
| The main security indicators | | Vital signs, laboratory indicators, adverse events (AEs), serious adverse events (SAEs) |  |

**1. Background**

**1.1 The effect of lymph node detection on the prognosis of gastric cancer**

Although the prevalence and mortality of gastric cancer have a gradual downward trend, its mortality rate is still ranked second in the world ^[1-2]^, China is in a high incidence of gastric cancer, the annual incidence of gastric cancer is 23.7/100,000, and the case fatality rate is 16.6/100,000.

Currently, surgical removal is the only cure for stomach cancer. Gastrectomy combined with standard lymphadenectomy is essential. Although standard lymph node dissection is considered a key procedure for radical resection of gastric cancer, there is no worldwide consensus on the number of lymph nodes detected after surgery. The number of lymph node dissections is affected by many factors: the degree of lymph node dissection, surgical skills, the patient's condition, the examination techniques used by the pathologist, etc. National Comprehensive Cancer Network (NCCN) guidelines recommend that the total number of postoperative lymph nodes should be at least 15 in order to accurately determine the N stage ^[3,4].^ The latest Japanese guidelines recommend that no fewer than 16 lymph nodes should be searched to accurately determine the N stage ^[5].^ Obviously, there is still controversy between different guidelines on how many lymph nodes should be removed. However, several studies have shown that more lymph node detection is an important factor in the quality control of lymph node dissection and correction of postoperative N stage deviation, and also affects the prognosis of patients ^[6-8].^

**1.2 Application of nanocarbon suspension injection in gastric cancer lymph node tracing**

In 2004, after the launch of China's self-developed nano-activated carbon (CNSI, Carnaline) (Chongqing Laimei Pharmaceutical Co., Ltd.), the price of nanocarbon was greatly reduced and medical costs were reduced. The nanocarbon suspension has a high lymphatic affinity and is made of polyvinylpyrrolidone and normal saline, wrapped in smooth carbon particles with an average aggregate diameter of 150 nm. After injection into the tissue around the tumor, it can be phagocytosed by the macrophage system in the body, and because the capillary basement membrane is more fully developed, the capillary endothelial cell space is 30-50nm, while the capillary lymphatic basement membrane is incomplete, the endothelial cell arrangement is shingled, and the endothelial cell space is 100-500nm. Therefore, CNSI can quickly enter capillary lymphatic vessels through macrophage phagocytosis and accumulate and remain in lymph nodes without entering blood vessels ^[9]^, which can play a good tracing effect on regional lymph nodes. In addition, activated carbon is black, more conspicuous, easier to identify during surgery, and has a strong staining ability for lymph nodes, long metabolic time, not easy to fade.

A number of reports suggest that nanocarbon has high safety, and no drug damage or related adverse reactions have been seen while increasing the detection rate of lymph nodes in radical gastric cancer resection. At the same time, the application of nanocarbon did not increase the operation time and postoperative complications of gastric cancer ^[10-12].^

**1.3** **Application of indocyanine green in gastric cancer lymph node tracing**

As a new surgical navigation technology, indocyanine green (ICG) near-infrared imaging technology has achieved positive results in sentinel lymph node dissection and localization of breast cancer, non-small cell lung cancer and other tumors.

In recent years, with the successful application of ICG fluorescence imaging technology in laparoscopic equipment, scholars have found that ICG near-infrared imaging has better tissue penetration, and can identify lymph nodes in hypertrophic adipose tissue better than other dyes under visible light, making ICG fluorescence imaging guided laparoscopic radical gastrectomy lymph node dissection a new exploration direction. In addition, ICG has been increasingly reported on organ reconstruction and gastrointestinal anastomotic blood vessel assessment. ICG near-infrared imaging technology has important research value, good application prospect and broad development space in laparoscopic radical gastric cancer surgery, and has attracted extensive attention and in-depth research at home and abroad. Several studies have shown that indocyanine green guided gastric lymph node dissection increases the number of lymph nodes detected after surgery, and obtains good clinical efficacy and safety ^[13-15].^

**2. The purpose, content and significance of the research**

**Main objective:** To evaluate the effect of nanocarbon and indocyanine green lymph node tracers on the number of lymph nodes detected after radical gastric cancer resection.

**Secondary objectives: Lymphatic staining (fluorescence) rate, lymph node staining rate** of each group, staining (fluorescence) lymph node positive rate, staining (fluorescence) lymph node negative rate, non-stained (fluorescent) lymph node positive rate, non-stained (fluorescent) lymph node negative rate, lymph node metastasis rate, number of lymph node metastasis, complication rate and mortality within 30 days, 3-year DFS, 3-year OS, 3-year recurrence pattern, intraoperative blood loss, postoperative lymph node sorting time, postoperative recovery (exhaust time, intake time, Time of discharge).

**Contents**: To evaluate the efficacy and safety of nanocarbon and indocyanine green-guided lymph node tracing technology in radical gastric cancer resection.

**Research Significance:** Through the randomized and controlled clinical study of nanocarbon versus indocyanine green-guided lymph node tracing technology in radical gastric cancer resection, the effect of different tracers on the postoperative pathological N stage and prognosis was further explored. Provide the best solution for lymph node tracing for radical surgery of gastric cancer.

**3. Implementation plan**

**Study design**

(1) Research Object:

Patients undergoing radical resection of gastric cancer.

(2) Research design

The study was a phase II. randomized, controlled clinical study, which included patients with gastric cancer who were pathologically confirmed to be adenocarcinoma by endoscopic bite examination, and clinically evaluated gastric cancer patients who were to undergo radical resection of gastric cancer, and all patients were randomly assigned to preoperative endoscopic submucosal injection of nanocarbon suspension injection or indocyanine green, drug selection and dose

CNSI group: 50mg/dose nanocarbon suspension produced by Chongqing Laimei Pharmaceutical. One day before surgery, nanocarbon labeling was received in the gastroscopy room, and nanocarbon was injected at four points (oral side, side, large curved side, and small curved side) at 0.5cm-1cm from the tumor edge endoscopically, and injected into the submucosal layer, and the amount of nanocarbon injected at each point was about 0.25ml;

ICG group: 25mg/dose of indocyanine green for injection produced by Dandong Yichuang Pharmaceutical. One day before surgery, indocyanine green was marked in the gastroscopy room, and indocyanine green was injected in 4 quadrants (oral side, side and left and right sides) at the junction of tumor boundary and normal mucosa, and injected into the submucosa with 0.5mL per point. Both procedures are performed by experienced physicians.

(3) Research indicators

Primary endpoint: Number of lymph nodes detected after surgery.

Secondary endpoints: lymphomatic staining (fluorescence) rate, lymph node staining rate by group, staining (fluorescence) lymph node positivity rate, staining (fluorescence) lymph node negative rate, non-staining (fluorescent) lymph node positive rate, non-stained (fluorescent) lymph node negative rate, lymph node metastasis rate, number of lymph node metastases, complication rate and mortality within 30 days, 3-year DFS, 3-year OS, 3-year recurrence pattern, intraoperative blood loss, postoperative lymph node sorting time, postoperative recovery (exhaust time, intake time, Time of discharge).

(4) Statistical methods

The sample size was calculated using PASS11 software; Estimates are based on the results of our center's previous research. The main calculation parameters of nanocarbon research:

Subjects will be randomized 1:1 to the CNSI group and the ICG group; Set the bilateral α=0.05 and β=0 8 0; The mean number of lymph nodes cleaned in the experimental group was expected to be 56.93, and the mean number of lymph nodes cleaned in the control group was 50.52. The standard deviation of the expected test group is 10. It was calculated that 96 subjects needed to be enrolled considering the 10% dropout rate. Among them, there were 48 cases in the C NSI group and 48 cases in the ICG group.

The mean, standard deviation, median, minimum and maximum values of the measurement data such as age, height, weight, and tumor clinicopathological data were calculated according to their distribution. The number and percentage of cases of each category were calculated for classification indicators such as sex and postoperative complications.

Comparison of metrological data between groups, univariate analysis using independent sample t-test or corresponding nonparametric test, screening and quantitative analysis of nanocarbon staining and other possible influencing factors. It was also analyzed whether nanocarbon staining had an impact on the operation time, blood transfusion volume, and postoperative complications.

All statistical analyses were performed using SPSS22.0, and all tests were two-sided with significance level α=0.05.

**Technical route**


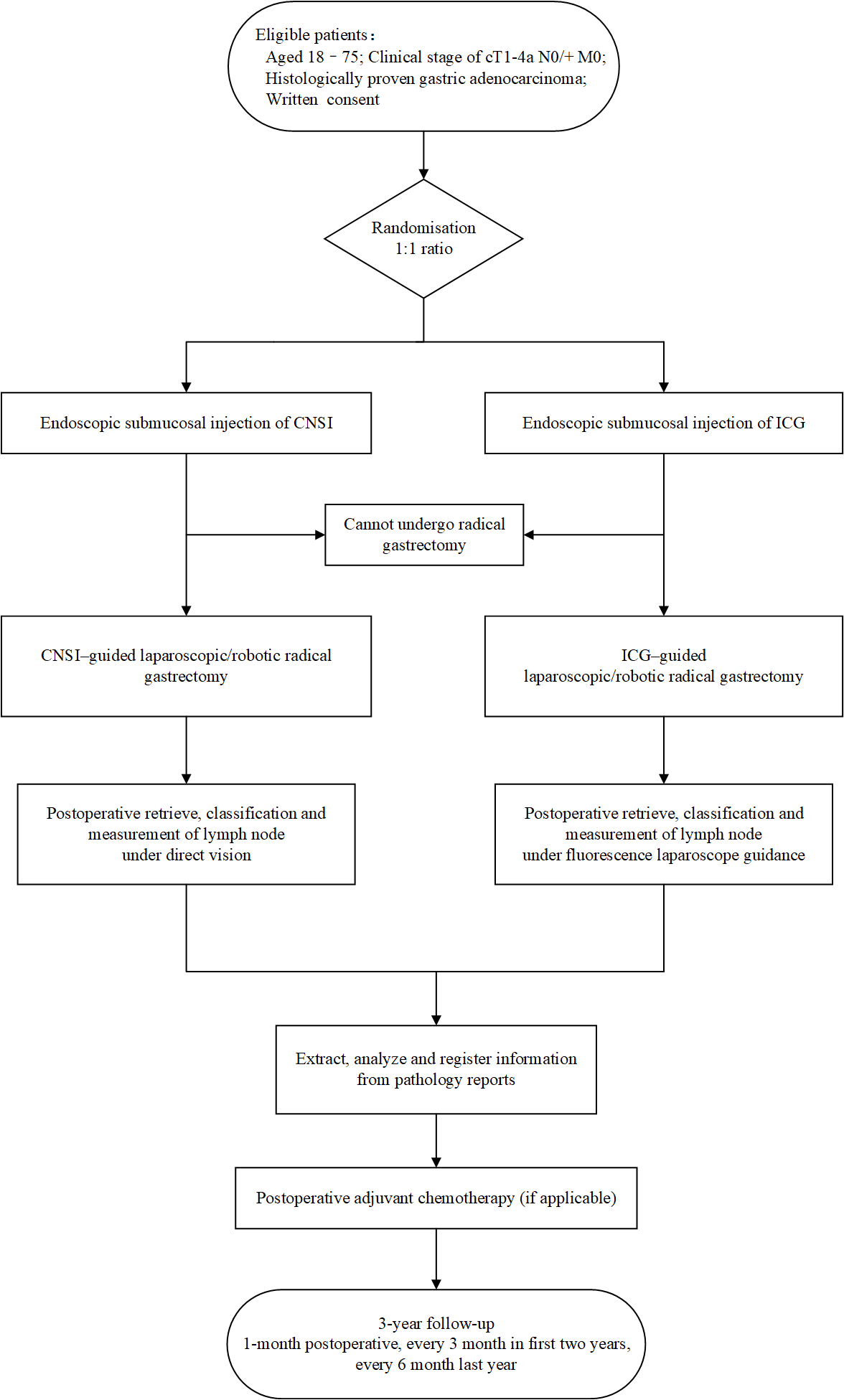


**Implementation steps**

(1) Inclusion criteria:

1. Age 18 to 75 years

2. Primary gastric adenocarcinoma confirmed by endoscopic biopsy pathology (papillary, tubular, mucinous, ring cell or hypodifferentiation)

3. According to the American Joint Committee on Cancer (AJCC) Cancer Staging Manual Eighth Edition, clinical stages cT1-4a, N 0/+, M0 at preoperative evaluation

4. No distant metastasis, no direct invasion of pancreas, spleen and other adjacent organs in preoperative examination.

5. Eastern Collaborative Oncology Group Scale (ECOG) 0 or 1 score

6. American Society of Anesthesia Score (ASA) GRADE I, II, or III

7. Sign a written informed consent form

(2) Exclusion criteria:

1. Women during pregnancy or lactation

2. Severe mental disorders

3. History of previous upper abdominal surgery (except laparoscopic cholecystectomy)

4. History of previous gastrectomy, endoscopic mucosal resection or endoscopic submucosal dissection

5. Preoperative imaging shows enlarged or enlarged regional lymph nodes with a diameter of more than 3 cm

6. Lesions invade the esophagus or duodenum

7. Borrmann type IV gastric cancer

8. History of other malignant tumors in the past five years

9. Previous history of neoadjuvant chemotherapy or radiotherapy

10. History of unstable angina or myocardial infarction within the past six months

11. History of cerebrovascular accident within the past six months

12. History of continuous and systematic use of corticosteroids within 1 month

13. Simultaneous surgical requirements for other diseases

14. Emergency surgery due to gastric cancer complications (bleeding, obstruction or perforation).

15.FEV1 < 50% of the predicted value.

(3) Screening and enrollment stages

1. Screening subjects who meet the criteria

2. Fully inform the patient or authorized person and obtain informed consent signed by the candidate subject or their guardian/legal representative. Randomize groupings.

(4) Treatment plan after enrollment

1. Endoscopic injection of lymph node tracer

CNSI group: 50mg/dose nanocarbon suspension produced by Chongqing Laimei Pharmaceutical. One day before surgery, nanocarbon labeling was received in the gastroscopy room, and nanocarbon was injected at four points (oral side, side, large curved side, and small curved side) at 0.5cm-1cm from the tumor edge endoscopically, and injected into the submucosal layer, and the amount of nanocarbon injected at each point was about 0.25ml;

ICG group: 25mg/dose of indocyanine green for injection produced by Dandong Yichuang Pharmaceutical. One day before surgery, indocyanine green was marked in the gastroscopy room, and indocyanine green was injected in 4 quadrants (oral side, side and left and right sides) at the junction of tumor boundary and normal mucosa, and injected into the submucosa with 0.5mL per point. Both procedures are performed by experienced physicians.

(v) Laparoscopic exploration:

Abdominal exploration (open or laparoscopic exploration). Exploration includes the following methods: palpation of abdominal organs and peritoneum, laparoscopic visual examination, and detection of free cancer cells in the abdominal cavity. ➀➁➂Rinse, agitate and collect well near the gastric cancer lesion with 8 00 mL of normal saline. Patients should be placed in a head-high and low-foot position, with 8 00 mL of normal saline injected from the right upper quadrant and at least 3 00 mL of flush fluid drawn from the pelvis in a head-high and low-foot position. After collecting the intraperitoneal irrigation solution, add 1 mL of heparin anticoagulation and centrifuge at 1000 g for 10 min. The nucleated cell layer was collected for smear, and tumor cells were detected by HE staining.

If there is no peritoneal implant metastasis and negative peritoneal exfoliation cytology, laparoscopic or robotic radical gastrectomy is performed.

(6) Lymph node dissection norms:

Patients enrolled in this study should be dissected with the D2 lymph node range specified in the Japanese gastric cancer protocol. The specific provisions are as follows:

Table 1 Extent of lymph node dissection for different sites of gastric cancer D1 and D2 (standard radical resection).

|  |  | Distal gastrectomy | Proximal gastrectomy | Total gastrectomy |
| --- | --- | --- | --- | --- |
|  |  |  |  |  |
|  | D1 | 1、3、4sb、4d、5、6、7 | 1、2、3、4sa、4sb、7 | 1-7 |
|  | D2 | D1+8a、9、11p、12a | D1+8a、9、10、11 | D1+8a、9、10、11、12a |
|  |  |  |  |  |

(7) Lymph node material specifications:

1) Time limit: lymph node collection should be started within half an hour after the specimen is isolated from the body and before formalin fluid fixation.

2) Subject: Lymph node extractors need to have experience in lymph node extraction, or complete it under the guidance of an experienced physician.

3) Lymph node grouping classification and examination: According to the naming method and anatomical markers stipulated in the Japanese gastric cancer protocol (Figure 1, 2), lymph nodes are sent separately according to group, staining or fluorescence, and whether they are ≤ 5mm.

Figure 1: Gastric cancer lymph node grouping

Figure 2: Anatomical diagram of gastric cancer lymph node grouping

4) Classification of lymph node examination group:

a. In patients in theCSI group, black-stained lymph nodes were sent separately from non-melanthema lymph nodes, such as a black-stained lymph node in group 6, which was sent for examination and identified as "group 6 black-stained lymph nodes".

b. In patients in the ICG group, fluorescent lymph nodes are sent separately from non-fluorescent lymph nodes, such as a fluorescent lymph node in group 6, which is labeled as "group 6 fluorescent lymph nodes".

(8) Pathology report:

The subject's pathology report should contain the following: tumor location, size, tissue type, degree of differentiation, Lauren classification, vascular cancer thrombus. Nerve invasion and immunohistochemistry. Lymph node pathology is reported separately according to the station sent by clinician.

(9) Surgical records:

Subjects should record and report the following contents: date of surgery, operation time (time from skin incision to abdominal closure), surgical method (laparoscopy, robot), gastric cancer surgery range (distal gastrectomy, total gastrectomy, proximal gastrectomy), lymph node dissection range, combined organ resection, digestive tract reconstruction method, intraoperative blood loss, etc.

(10) Surgical complications:

1) Intraoperative complications: bleeding, organ damage, lymphatic leakage, etc.

2) Postoperative complications: Complications during the hospitalization of subjects after surgery were recorded and graded according to the Clavien-Dindo complication grading system (Table 2).

| **Clavien-Dindo complication grading** | |
| --- | --- |
| grading | description |
| Class I | Any complications that deviate from the natural course after surgery, including the use of antiemetics, antipyretics, analgesics, diuretics, fluids, and physiotherapy, as well as incision infections in bedside debridement |
| Class II | Pharmacologic therapy other than those permitted for grade I complications is required, including blood transfusion and total parenteral nutrition support |
| Grade III | Requires surgical, endoscopic, and interventional radiotherapy |
| Class III.a | General anesthesia is not required |
| Class III.b | General anesthesia is required |
| Class IV | Life-threatening complications, including central nervous system complications, require intermittent monitoring or intensive care unit (ICU) treatment |
| Class IV.a | 1 organ insufficiency (including dialysis). |
| Class IV.b | Multiple organ insufficiency |
| Class V | death |

Table 2: Clavien-Dindo complication grading

(11) Surgery, lymph node material control:

The participating center of this study needs to provide 4 screenshots of the enrolled patients after lymph node dissection and 1 photo of the postoperative pathology report.

1. Pyloric area: contains the right gastric and right gastroomentum vascular discontinuation
2. Suprapancreatic area: contains the severed end of the left blood vessel of the stomach
3. Splenic area: including the severed end of the left blood vessel of the gastrooretina, (if splenic lymph node dissection, also the spleen)
4. Small curved side of cardia: Screenshot of the small curved side of cardia

(12) Ethical requirements and informed consent of subjects

Written consent from the ethics committee is required prior to the start of the trial. Research must comply with the Declaration of Helsinki and Chinese norms and regulations on clinical research. Before subjects are enrolled in the study, the investigator should fully inform the subject or the subject's legal representative about the purpose, procedure, and possible risks of the trial. Participants are informed of their right to withdraw from the trial at any time and under any circumstances. Selected subjects must sign an informed consent form. Investigators must properly keep all documents approved by the ethics committee, including patients' informed consent forms, recruitment materials, etc. for monitoring.

(13) Progress and completion time of clinical trials

Case collection and treatment were completed within 1 year, and case follow-up was completed within 3 years.

(14) Preservation of materials

All research data and original records in the course of clinical research should be preserved in their entirety. The research unit shall retain these materials for 5 years after the end of the study. All data in this study were kept by the Fourth Hospital of Hebei Medical University.

**References**

1. Organization WH: Cancer, Fact sheet N°297, World Health Organization Media centre, Published 2009. Reviewed January 2013.
2. Jemal A, Siegel R, Xu J, et al: Cancer statistics, 2010. CA Cancer J Clin 60:277-300,2010
3. Ajani JA, D'Amico TA, Almhanna K, Bentrem DJ, Chao J, Das P, et al. Gastric cancer, version 3.2016, NCCN clinical practice guidelines in oncology. J Natl Compr Cancer Netw. 2016; 14(10):1286–1312. doi: 10.6004/jnccn.2016.0137.
4. Washington K. 7th edition of the AJCC cancer staging manual: stomach. Ann Surg Oncol. 2010; 17(12):3077–3079. doi: 10.1245/s10434-010-1362-z.
5. Japanese Gastric Cancer Association. Japanese gastric cancer treatment guidelines 2018 (5th edition). Gastric Cancer. 2021 Jan; 24(1):1-21. doi: 10.1007/s10120-020-01042-y. Epub 2020 Feb 14. PMID: 32060757; PMCID: PMC7790804.
6. Michele Orditura,Gennaro Galizia,Vincenzo Sforza, et al;. Treatment of gastric cancer[J]. World Journal of Gastroenterology,2014,20(07):1635-1649.
7. Chinese guidelines for diagnosis and treatment of gastric cancer 2018(English version)[J]. Chinese Journal of Cancer Research,2019,31(05):707-737.
8. Guangchuan Mu,Yuan Huang,Chengzhi Wei, et al; Para-aortic lymph node tracing and dissection in advanced gastric cancer: Effectiveness of carbon nanoparticles injection through the no. 12b lymph node[J]. Journal of Cancer Research and Therapeutics,2020,16
9. Lu Y,Wei J Y,Yao D S,et al. Application of carbon nanoparticles in laparoscopic sentinel lymph node detection in patients with early-stage cervical cancer[J]. Journal of International Obstetrics & Gynecology, 2016,12(9):e0183834.
10. TIAN Yuan,LIN Yecheng,LI Yong,FAN Liqiao,ZHANG Zhidong,WANG Dong,ZHAO Xuefeng,YANG Peigang,ZHAO Qun. Application value of nanocarbon in robot-assisted radical gastric cancer resection[J].Chinese Journal of Minimally Invasive Surgery,2021,(7):610-614.)
11. ZHANG Zhidong,LIU Qingwei,LI Yong,ZHAO Qun,FAN Liqiao,JIAO Zhikai,ZHAO Xuefeng,WANG Dong,LIU Yu. Application value of nanocarbon in lymph node detection after preoperative chemotherapy for locally advanced gastric cancer[J].Chinese Journal of General Practice,2016,19(2):179-183.)
12. ZHANG Zhidong,LIU Qingwei,LI Yong,ZHAO Qun,FAN Liqiao,TAN Bibo,WANG Dong,ZHAO Xuefeng,JIAO Zhikai,LIU Yu. Application of nanocarbon lymphatic tracer in radical gastric resection of early gastric cancer[J].Guangdong Medical Journal,2015,36(17):2698-2701.)
13. Kwon In Gyu, Son Taeil, Kim Hyoung-Il, et al. Fluorescent Lymphography-Guided Lymphadenectomy During Robotic Radical Gastrectomy for Gastric Cancer.. JAMA Surgery, 2019, 154(2):150-158.
14. Assessment of the Completeness of Lymph Node Dissection Using Near-infrared Imaging with Indocyanine Green in Laparoscopic Gastrectomy for Gastric Cancer. Journal of Gastric Cancer, 2018, 18(2):161-171.
15. Chen QY,Xie JW,Zhong Q, et al. Safety and Efficacy of Indocyanine Green Tracer-Guided Lymph Node Dissection During Laparoscopic Radical Gastrectomy in Patients With Gastric Cancer: A Randomized Clinical Trial. [J]. JAMA surgery,2020.
