# Supplementary material for: Effect of carbon nanoparticle suspension injection versus indocyanine green tracer in guiding lymph node dissection during radical gastrectomy (FUTURE-01): a randomized clinical trial
Source: Int J Surg. 2024 Jul 2;111(1):609–16. doi: 10.1097/JS9.0000000000001873 (PMC11745718; doi:10.1097/JS9.0000000000001873)
Supplement: Supplementary file 9 [file js9-111-0609-s009.docx]

Table 1. Metastatic lymph nodes (LNs) in the carbon nanoparticle suspension injection (CNSI) and indocyanine green (ICG) groups

| Metastatic LNs | CNSI (n=46) | ICG (n=44) | P value |
| --- | --- | --- | --- |
| No. 1 | 0.2±1.2 | 0.4±0.9 | 0.393 |
| No. 2 | 0.3±1.2 | 0.1±0.3 | 0.201 |
| No. 3 | 2.6±4.9 | 1.5±2.8 | 0.211 |
| No. 4sa | 0.2±0.7 | 0.1±0.4 | 0.504 |
| No. 4sb | 0.7±2.2 | 0.2±0.6 | 0.096 |
| No. 4d | 1.4±3.2 | 0.7±1.7 | 0.195 |
| No. 5 | 0.3±0.8 | 0.5±1.9 | 0.565 |
| No. 6 | 0.8±2.1 | 0.4±1.1 | 0.261 |
| No. 7 | 1.1±2.3 | 0.7±1.5 | 0.299 |
| No. 8a | 0.2±0.6 | 0.1±0.4 | 0.879 |
| No. 9 | 0.1±0.4 | 0.2±0.7 | 0.249 |
| No. 11p | 0.1±0.6 | 0.3±1.2 | 0.301 |
| No. 11d | 0 | 0 | 0 |
| No. 12a | 0.1±0.7 | 0 | 0.244 |
