# Supplementary material for: Effect of carbon nanoparticle suspension injection versus indocyanine green tracer in guiding lymph node dissection during radical gastrectomy (FUTURE-01): a randomized clinical trial
Source: Int J Surg. 2024 Jul 2;111(1):609–16. doi: 10.1097/JS9.0000000000001873 (PMC11745718; doi:10.1097/JS9.0000000000001873)
Supplement: Supplementary file 10 [file js9-111-0609-s010.docx]

**Table 2.** Diagnostic value of carbon nanoparticle suspension injection (CNSI)- and indocyanine green (ICG)-guided lymphography for detecting metastatic lymph nodes (LNs) based on the total number of LNs

|  | Total no. | No. of metastatic LNs | Number of nonmetastatic LNs | Sensitivity  (%) | Specificity  (%) | PPV  (%) | NPV  (%) |
| --- | --- | --- | --- | --- | --- | --- | --- |
| CNSI |  |  |  |  |  |  |  |
| Stained | 2123 | 269 (TP) | 1854 (FP) | 72.1 | 34.6 | 12.7 | 90.4 |
| Nonstained | 1087 | 104 (FN) | 983 (TN) |  |  |  |  |
| ICG |  |  |  |  |  |  |  |
| Stained | 1222 | 98 (TP) | 1124 (FP) | 42.8 | 47.3 | 8 | 88.5 |
| Nonstained | 1138 | 131 (FN) | 1007 (TN) |  |  |  |  |

*LNs, lymph nodes; PPV, positive predictive value; NPV, negative predictive value; TP, true positive; FP, false positive; FN, false negative; TN, true negative.
