# Supplementary material for: Effect of carbon nanoparticle suspension injection versus indocyanine green tracer in guiding lymph node dissection during radical gastrectomy (FUTURE-01): a randomized clinical trial
Source: Int J Surg. 2024 Jul 2;111(1):609–16. doi: 10.1097/JS9.0000000000001873 (PMC11745718; doi:10.1097/JS9.0000000000001873)
Supplement: Supplementary file 11 [file js9-111-0609-s011.docx]

**Table 3.** Diagnostic value of carbon nanoparticle suspension injection (CNSI)- and indocyanine green (ICG)-guided lymphography for the detection of metastatic micro-lymph nodes (micro-LNs)

|  | Total no. | No. of metastatic micro-LNs | Number of nonmetastatic micro-LNs | Sensitivity  (%) | Specificity  (%) | PPV  (%) | NPV  (%) |
| --- | --- | --- | --- | --- | --- | --- | --- |
| CNSI |  |  |  |  |  |  |  |
| Stained | 587 | 44 (TP) | 543 (FP) | 70.9 | 36.6 | 7.5 | 94.6 |
| Nonstained | 331 | 18 (FN) | 313 (TN) |  |  |  |  |
| ICG |  |  |  |  |  |  |  |
| Stained | 252 | 13 (TP) | 239 (FP) | 40.6 | 50.2 | 5.2 | 92.7 |
| Nonstained | 260 | 19 (FN) | 241 (TN) |  |  |  |  |

*LNs, lymph nodes; PPV, positive predictive value; NPV, negative predictive value; TP, true positive; FP, false positive; FN, false negative; TN, true negative.
