# Supplementary figures and images for: Effect of carbon nanoparticle suspension injection versus indocyanine green tracer in guiding lymph node dissection during radical gastrectomy (FUTURE-01): a randomized clinical trial
Source: Int J Surg. 2024 Jul 2;111(1):609–16. doi: 10.1097/JS9.0000000000001873 (PMC11745718; doi:10.1097/JS9.0000000000001873)

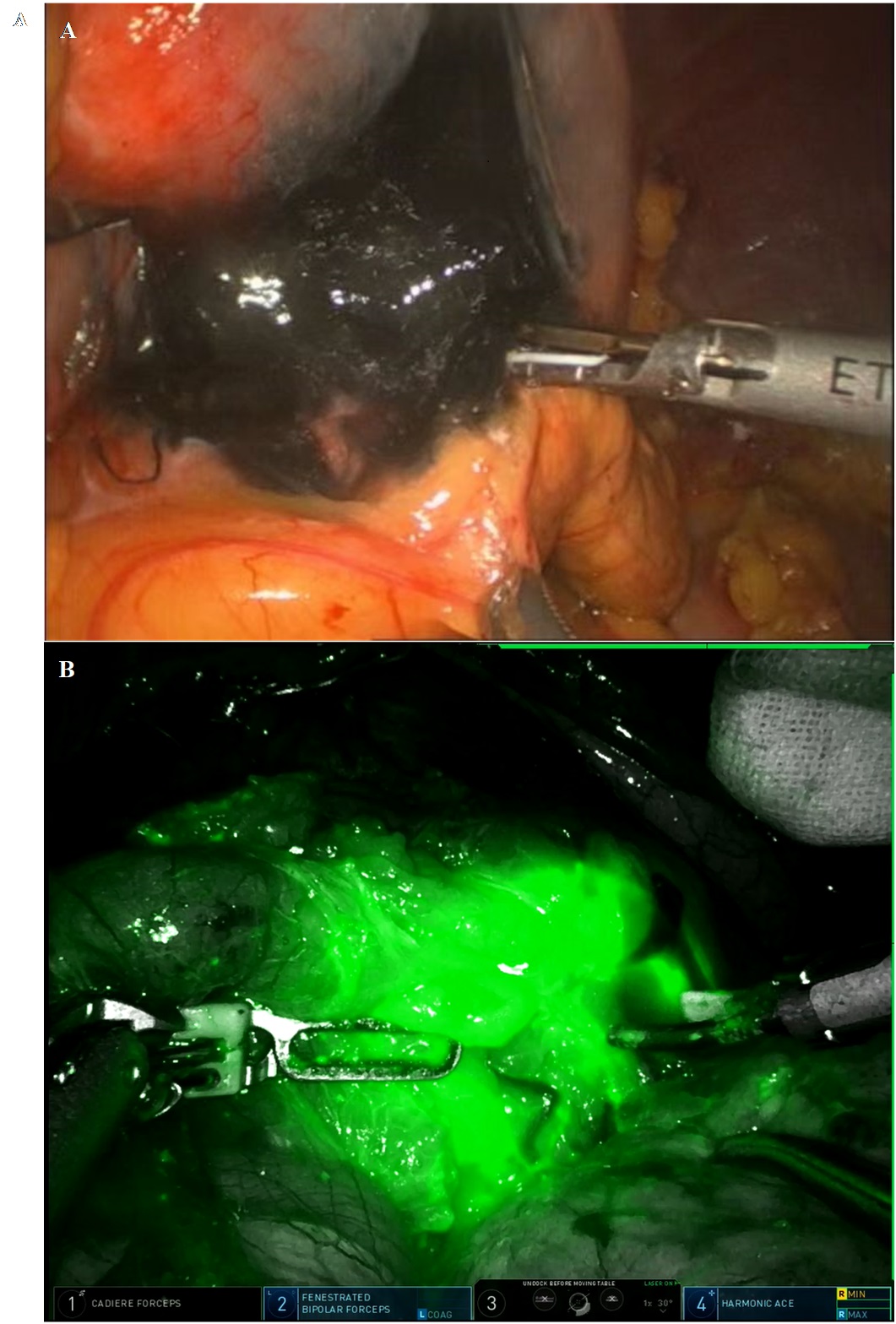

Supplement: Supplementary file 12 [file js9-111-0609-s012.jpg]
